# A range of voltage-clamp protocol designs for rapid capture of hERG kinetics

**DOI:** 10.12688/wellcomeopenres.23319.2

**Published:** 2025-07-09

**Authors:** Chon Lok Lei, Dominic J Whittaker, Monique J Windley, Matthew D Perry, Adam P Hill, Gary R Mirams

**Affiliations:** 1 University of Macau Institute of Translational Medicine, Taipa, Macao; 2 University of Macau Department of Biomedical Sciences, Taipa, Macao; 3 University of Nottingham School of Mathematical Sciences, Nottingham, England, NG7 2RD, UK; 4 Computational Cardiology Laboratory, Victor Chang Cardiac Research Institute, Darlinghurst, New South Wales, Australia; 5 University of New South Wales School of Clinical Medicine, Sydney, New South Wales, Australia; 6 University of New South Wales School of Biomedical Sciences, Sydney, New South Wales, Australia

**Keywords:** experimental design, ion channel, mathematical model, computational model, patch clamp

## Abstract

We provide details of a series of short voltage-clamp protocols designed for gathering a large amount of information on hERG (K _v_11.1) ion channel gating. The protocols have a limited number of steps and consist only of steps and ramps, making them easy to implement on any patch clamp setup, including automated platforms. The primary objective is to assist with parameterisation, selection and refinement of mathematical models of hERG gating. We detail a series of manual and automated model-driven designs, together with an explanation of their rationale and design criteria. Experimental data under all these protocols is available in a partner publication. Although the protocols are intended to study hERG1a currents, the approaches could be easily extended and generalised to other ion channel currents.

## Plain language summary

Ion channels are proteins that span the membranes of biological cells, and they allow certain ions to flow through them, to cross the membrane (e.g. K ^+^, Na ^+^ or Ca ^2+^). They are important in controlling concentrations of ions within the cell, and also used by the body to transmit electrical signals - controlling processes like nerve impulses or co-ordination of muscle contraction such as in the heart.

Many ion channels open and close in response to changes in the voltage across the cell membrane. Working out the intricate details of exactly how ion channels respond to voltage is difficult and often time consuming. A good method to use is voltage clamping, where some electronic apparatus is cleverly attached to a cell such that there is effectively one electrode inside the cell and one outside. By clamping the membrane voltage to carefully chosen waveforms, and recording the currents that flow, we can investigate how currents respond to voltage.

It is an active topic of research deciding what voltage waveform is most effective to use. One recently proposed route is to use comparatively short waveforms that rapidly perturb voltage, and use the resulting data to fit a mathematical model of the ion channel opening and closing. This article proposes a whole suite of this type of waveform, some designed manually and some designed by mathematical algorithms, which we expect to be valuable in characterising a particular heart ion channel current (hERG). The protocols should provide abundant data to train and test mathematical models of the current. These models can then be used to communicate how we think the channel works and to make predictions of its behaviour in new situations.

## Introduction

This report describes a series of voltage-clamp protocol waveforms that were designed to explore the gating of cell lines expressing hERG1a / K _v_11.1 channels, which are the primary subunit of the channels carrying the cardiac rapid delayed rectifier potassium current, I _Kr_ ( Sanguinetti *et al.*, 1995; Vandenberg *et al.*, 2012).

The aim is to build on our previous studies that aimed to develop a range of short, information-rich voltage clamp protocols to use in experimental recordings to capture hERG gating behaviour ( Beattie *et al.*, 2018; Lei *et al.*, 2019b). Here we extend these to a wide range of protocols to better parameterise, select and test mathematical models of hERG gating ( Bett *et al.*, 2011) and in particular to gain a better understanding and quantification of model discrepancy — when models cannot correctly predict what happens in reality ( Shuttleworth *et al.*, 2024). As a result, some protocols will focus on classic optimal experimental design in terms of reducing uncertainty / improving identifiability of model parameter estimates ( Lei *et al.*, 2023). Whilst others focus on maximising differences between trained models to assist in model selection/discrimination.

All these protocols were designed during the Isaac Newton Institute’s Fickle Heart programme in May–June 2019 ( Mirams *et al.*, 2020). The protocols are all designed to be run on an automated patch platform, namely the Nanion SyncroPatch384PE ( Obergrussberger *et al.*, 2016), which at the time had a restriction of only allowing up to 64 commands (steps or ramps) to define a single voltage-clamp protocol. Experimental data from a hERG1a CHO cell expression system at room temperature for these protocols was published in Shuttleworth *et al*. (2025).

## Models used in protocol design process

Our designs are model-driven akin to Lei *et al.* (2023), where mathematical models are used as part of automatic optimal design; even where our designs are manual they were done by visually examining the results of forward simulations.

**Figure 1.**
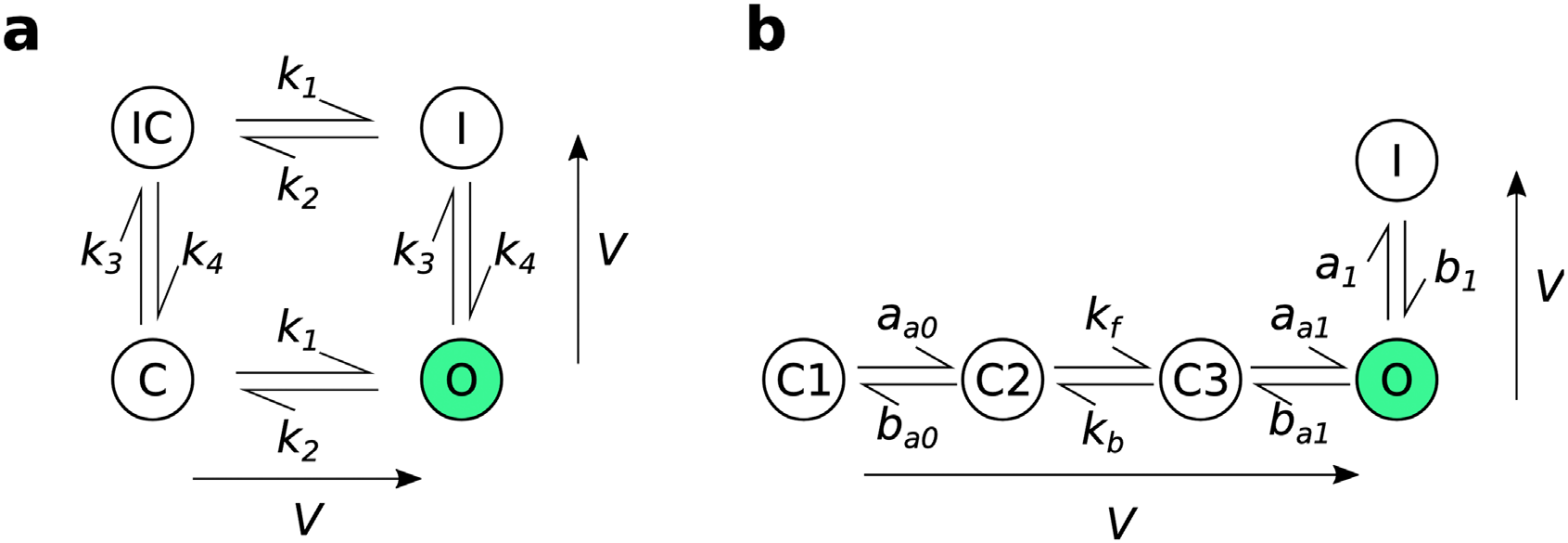
The model structures used for experimental design. ( **a**): the four-state Beattie *et al.* (2018) model. ( **b**): the five-state Wang *et al.* (1997) model. The arrows adjacent to each model structure indicate the direction in which rates increase as the voltage increases. Reproduced from Shuttleworth *et al.* (2024) under a CC-BY licence.

The model structures that we used here are Beattie *et al.* (2018) and Wang *et al.* (1997) (also used in Fink *et al.* (2008)), with their Markov diagrams shown in Figure 1 and full equations reproduced below. The first model ( Beattie *et al.*, 2018) is a Hodgkin-Huxley style model with two independent gates, which can be represented as a symmetric 4-state Markov model (see Fig. 4B of Rudy and Silva (2006)). The second model Wang *et al.* (1997) is a 5-state Markov model with 3 closed states, an open state, and an inactivated state connected sequentially.

### Beattie model

In matrix/vector form, the Beattie *et al.* (2018) model can be written as,


$$\frac{\text{d}\text{x}}{\text{d}t}=\left[\begin{array}{cccc} -k_{1}-k_{3} & 0 & k_{4} & k_{2}\\ 0 & -k_{2}-k_{4} & k_{1} & k_{3}\\ k_{3} & k_{2} & -k_{1}-k_{4} & 0\\ k_{1} & k_{4} & 0 & -k_{2}-k_{3} \end{array}\right]\;\text{x},$$

where


$$\text{x}={\left[C,\;I,\;IC,\;O\right]}^{T},$$

and


$$\begin{array}{l} k_{1}\;=p_{1}e^{p_{2}V},\\ k_{2}\;=p_{3}e^{-p_{4}V},\\ k_{3}\;=p_{5}e^{p_{6}V},\\ k_{4}\;=p_{7}e^{-p_{8}V}. \end{array}$$

This model is equivalent to a two gate Hodgkin-Huxley style gating model with open probability given by an “activation” *a* gate representing the ‘right’ transitions in Figure 1a multiplied by an “inactivation” *r* gate representing the ‘down’ transitions ( Clerx *et al.*, 2019a; Mirams, 2023), so in the below designs when we refer to “Hodgkin-Huxley” (HH) it is this interpretation of the model we are using.

### Wang model

The Wang *et al.* (1997) model can be written as:


$$\frac{\text{d}\text{x}}{\text{d}t}=[\begin{array}{ccccc} -a_{a0} & b_{a0} & 0 & 0 & 0\\ a_{a0} & -b_{a0}-k_{f} & k_{b} & 0 & 0\\ 0 & k_{f} & -k_{b}-a_{a1} & b_{a1} & 0\\ 0 & 0 & a_{a1} & -b_{a1}-a_{1} & b_{1}\\ 0 & 0 & 0 & a_{1} & -b_{1} \end{array}]\;\text{x}\text{,}$$

where


$$\text{x}={\left[C_{1},\;C_{2},\;C_{3},\;O,\;I\right]}^{T},$$

and


$$\begin{array}{c} a_{1}\;=q_{1}e^{q_{2}V},\\ a_{a0}=q_{3}e^{q_{4}V},\\ a_{a1}=q_{5}e^{q_{6}V},\\ \;b_{a1}=q_{7}e^{-q_{8}V},\\ \;b_{1}=q_{9}e^{-q_{10}V},\\ \;b_{a0}=q_{11}e^{-q_{12}V}. \end{array}$$

The default (room temperature) parameter values for both models are presented in Table 1. In practice we remove one state from the system and set it equal to “one minus the sum of the rest” to solve the ODE system, to improve numerical stability. All models are solved using a Python package Myokit ( Clerx *et al.*, 2016) using SUNDIALS CVODE ( Hindmarsh *et al.*, 2005).

**Table 1.** The default parameter sets we use for the Wang *et al.* (1997) and Beattie *et al.* (2018) models. The column ‘Range’ indicates the parameter range obtained from real data fitting results based on protocols staircaseramp, sis, hh3step, and wang3step, which is used for global sensitivity-based designs.

Wang model				Beattie Model			
	Value	Range	Units		Value	Range	Units
*g*	2.11	—	×10 ^–1^ *μ*S	*g*	2.44	—	×10 ^–1^ *μ*S
*k _b_*	0.67	[0.67,99993]	×10 ^–2^ ms ^–1^	*p*_1_	1.68	[1.39,12.9]	×10 ^–4^ ms ^–1^
*k _f_*	1.31	[1.31,99550]	×10 ^–2^ ms ^–1^	*p*_2_	8.06	[1.08,8.49]	×10 ^–2^ mV ^–1^
*q*_1_	1.24	[1.24,1.81]	×10 ^–1^ ms ^–1^	*p*_3_	4.34	[2.77,32.3]	×10 ^–5^ ms ^–1^
*q*_2_	1.56	[1.55,2.06]	×10 ^–2^ mV ^–1^	*p*_4_	4.07	[2.48,4.56]	×10 ^–2^ mV ^–1^
*q*_3_	0.04	[0.03,1.02]	×10 ^–2^ ms ^–1^	*p*_5_	9.07	[6.40,19.9]	×10 ^–2^ ms ^–1^
*q*_4_	10.9	[0.0001,10.9]	×10 ^–2^ mV ^–1^	*p*_6_	2.67	[2.18,3.87]	×10 ^–2^ mV ^–1^
*q*_5_	0.24	[0.23,364]	×10 ^–2^ ms ^–1^	*p*_7_	7.32	[7.07,10.9]	×10 ^–3^ ms ^–1^
*q*_6_	0.0001	[0.0001,6.44]	×10 ^–2^ mV ^–1^	*p*_8_	3.22	[2.89,3.39]	×10 ^–2^ mV ^–1^
*q*_7_	3.15	[1.29,7.69]	×10 ^–4^ ms ^–1^				
*q*_8_	3.99	[2.97,3.99]	×10 ^–2^ mV ^–1^				
*q*_9_	5.75	[3.55,5.75]	×10 ^–3^ ms ^–1^				
*q*_10_	2.89	[2.89,3.34]	×10 ^–2^ mV ^–1^				
*q*_11_	0.28	[0.007,1458]	×10 ^–2^ ms ^–1^				
*q*_12_	10.7	[1.16,11.8]	×10 ^–2^ mV ^–1^				

## Common protocol segments

As described in Mirams *et al.* (2024), all the protocols we have designed have common start and end segments, as defined in Table 2. The purposes of these segments are:

**Table 2.** Details of the Start and End clamp sections for all designs. ‘t’ indicates the duration of the clamp section, and ‘V’ the relevant voltage(s) for this clamp. Where ‘Ramp’ is specified it is a linear ramp over time between the voltages shown, as opposed to a constant voltage clamp for a ‘Step’. Reproduced from Mirams *et al.* (2024).

Clamp	Initial: for leak and conductance			End: reversal ramp sequence		
#	Step/Ramp	t (ms)	V (mV)	Step/Ramp	t (ms)	V (mV)
1	Step	250	–80	Step	1000	–80
2	Step	50	–120	Step	500	40
3	Ramp	400	–120 to –80	Step	10	–70
4	Step	200	–80	Ramp	100	–70 to –110
5	Step	1000	40	Step	390	–120
6	Step	500	–120	Step	500	–80
7	Step	1000	–80	—	—	—

- Start — a leak ramp from -120 to -80 where hERG should be closed and we can estimate leak properties, followed by an ‘activation step’ to provoke a very large tail current and help with conductance estimation, as discussed in Beattie *et al.* (2018).
- End — a ‘reversal ramp’ to help assess whether the current is reversing at the expected Nernst potential, discussed in Lei *et al.* (2019b). Followed by 390ms at -120mV to ensure channel closing at the end of the protocol, so that the initial channel state for the next protocol does not depend on this preceding one.
- both can also be used in quality control to check that these sections behave similarly over time when different protocols are applied to the same cell.

## Manual protocol designs

The details of the protocols in this section are provided in the Supplementary Spreadsheet, note that all of them have the common protocol segments from Table 2 added.

### Original staircase protocol

**Figure 2.**
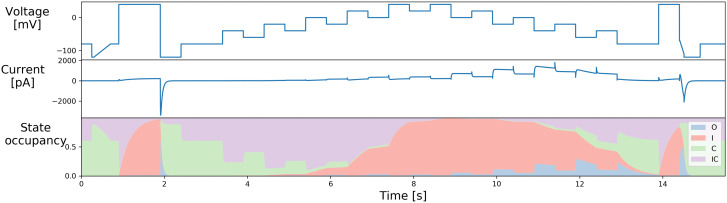
The manually-designed staircase protocol used in Lei, Clerx, Beattie, *et al.* (2019a; Lei *et al.*, 2019b) and its simulation, with state occupancy shown for the Beattie *et al.* (2018) model of Figure 1a. Reproduced from ( Lei *et al.*, 2019b) under a CC-BY licence.

Figure 2 shows the original staircase protocol. It was manually designed to capture various dynamics of hERG ( Lei *et al.*, 2019a; Lei *et al.*, 2019b), which has been used and tested on the Nanion SyncroPatch384PE. We have been using it as a quality control of the full run of the experiments when designing the protocols in the rest of this report.

### Staircase-in-staircase protocol

**Figure 3.**
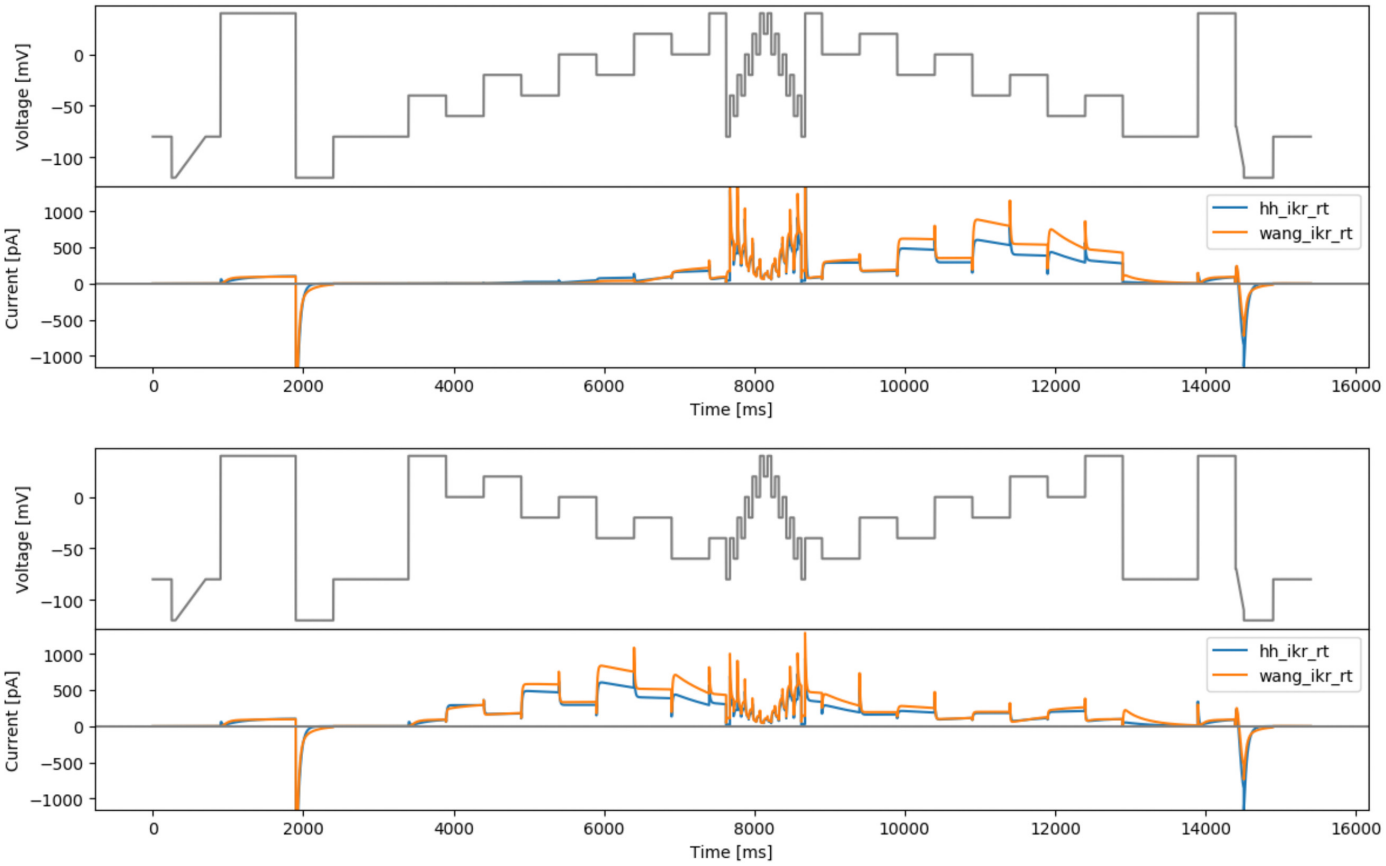
Manual designs. Top: the staircase-in-staircase (sis) protocol. Bottom: the ‘inverted’ staircase-in-staircase (sisi) protocol. Underneath each protocol are simulated currents from the two models ( hh_ikr_rt is the Beattie *et al.* (2018) model of I _Kr_ and wang_ikr_rt is the Wang *et al.* (1997) model of I _Kr_, both parameterised to room temperature data).

The original staircase protocol provided a good foundation and motivation for improving experimental designs for characterisation of ion channel kinetics in high-throughput machines. We attempted to further improve this manual design by enhancing the exploration of inactivation processes of hERG. The original staircase protocol involves only voltage steps of 500 ms, which may not be able to explore fully the fast dynamics of hERG inactivation processes. Therefore, a shorter step duration version (50 ms) of the full staircase protocol is introduced at the middle of the staircase protocol, termed the staircase-in-staircase (sis) protocol ( Figure 3, top). We also explored the possibility of inverting the order of the staircase as shown in Figure 3, bottom (sisi).

### Phase-space filling protocol

**Figure 4.**
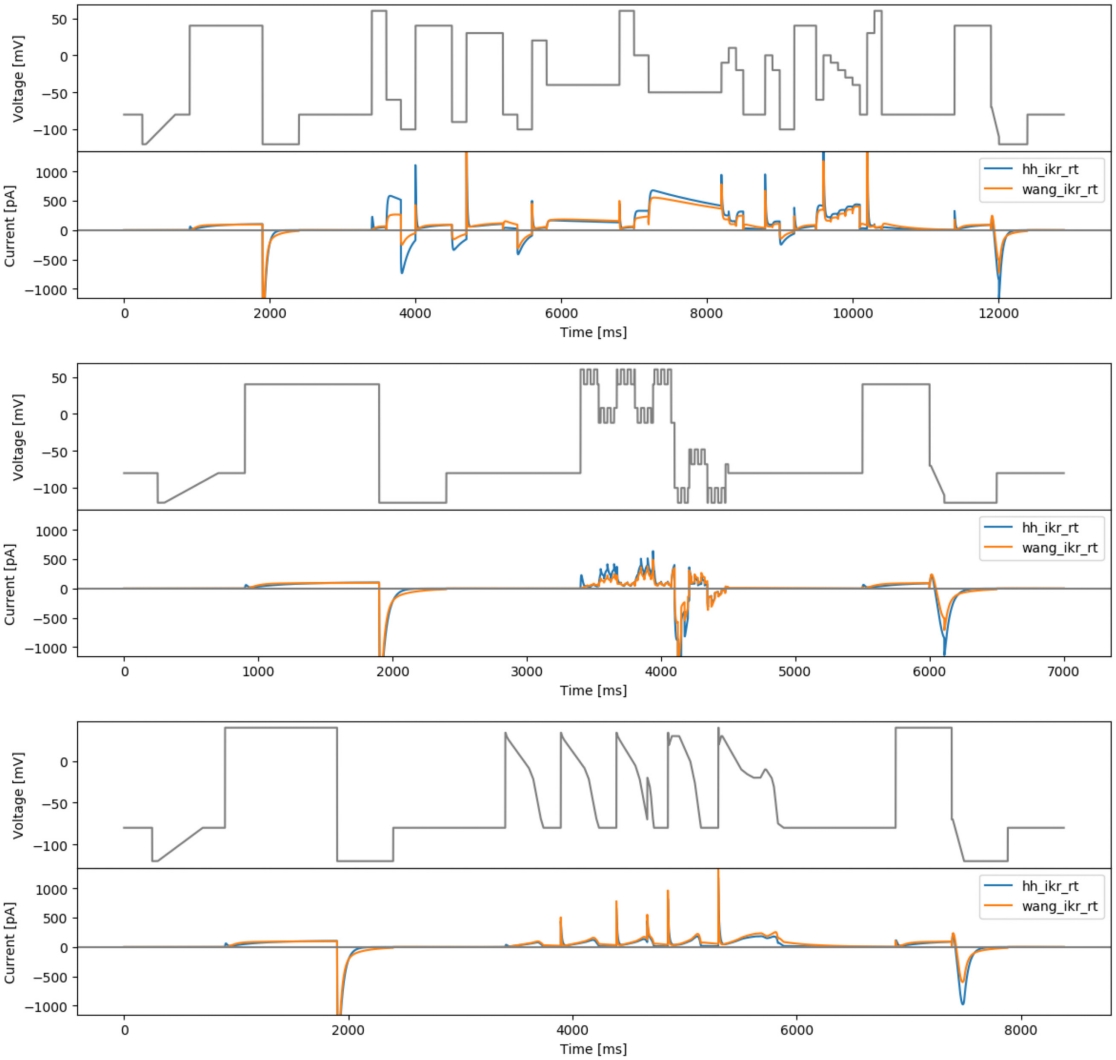
More manual designs. Top: the manual phase space protocol (manualppx). Middle: the square wave protocol of Beattie *et al.* (2018) (squarewave). Bottom: the lumped action potential protocol (longap). Beneath each protocol we show simulated currents from both the Beattie and Wang models.

The idea here is to have a protocol that fills up the phase-voltage space as much as possible. In brief, this design draws out the *a*, *r*, *V* three dimensional ‘phase-voltage space’ {[0,1],[0,1],[-120,60]} for the Beattie *et al.* (2018) model and subdivides it into 6 compartments in each dimension, giving a total of N = 6 ^3^ = 216 boxes. Since the phase space defines all possible behaviours of a model, if a protocol forces the model to visit as many of these boxes as possible, then the observations should test model assumptions well and provide rich information to fit model parameters. We have published the rationale and details of the design process for these protocol separately in Mirams *et al.* (2024). Figure 4 (top) shows a manually-tuned phase space filling protocol (manualppx); no objective function *per se*.

### A square-wave conversion of the sinusoidal protocol

In this design, we aim to design protocols based on sums of square waves, as inspired by Beattie *et al.* (2018). Such a protocol consists of a combination of *N* square waves, where each square wave *i* is defined by amplitude *a _i_*, (angular) frequency *ω _i_*, and phase lag *ϕ _i_*. The protocol is defined by 3 *N* parameters plus an extra parameter for an offset voltage, which can be expressed as:


$$V_{\text{square}\;\text{wave}}(t)=b+\sum_{i}^{N}a_{i}\;\text{sign}(\sin(\omega_{i}t+\phi_{i})),\;(1)$$

where the function sign(⋅) takes a value +1 if its argument is positive, -1 if negative, or 0 if the argument is 0.

A direct conversion of the sine waves in the Beattie *et al.* (2018) protocol is performed, with the same amplitudes and frequencies, to square waves. It is a combination of three square waves ( *N* = 3) with *a*_1_ = 54 mV, *a*_2_ = 26 mV, *a*_3_ = 10 mV, *ω*_1_ = 0.007 ms, *ω*_2_ = 0.037 ms, *ω*_1_ = 0.19 ms, and *ϕ*_1_ = *ϕ*_2_ = *ϕ*_3_ = 0, and an offset of *b* = −30 mV. The resulting protocol is called ‘squarewave’ and is shown in Figure 4 (middle).

### Long action potential protocol

As a final ‘manually-chosen’ design, we also propose a lumped action potential protocol for validation purposes, as shown in Figure 4 (bottom). It consists of two action potential morphologies, an early after-depolarisation (EAD)-like action potential, and a delayed after-depolarisation (DAD)-like action potential.

## Automated Iterative 3-step designs

Here we describe protocol design approaches that can be done objectively and automatically. With the same rationale as described in Mirams *et al.* (2024), we consider a protocol consists of 3 *N* steps with *N* ∈ ℕ, and we split the protocol into *N* units with 3 consecutive voltage steps as a unit. For some designs, *N* is the number of model parameters, while for others, *N* is 17 to bring the total number of steps to 51 which is close to the 64 allowed by the Nanion SyncroPatch384PE when the start and end clamps are added ( Table 2). For each unit *i*, we optimise the 3 voltage steps through an objective function *S _i_*, with each step defined by two parameters: voltage *V* and duration *Δt*. Each objective function *S _i_* (described in the sections below) aims to achieve a different purpose. We then iterate the process for all the objective functions *i* = 1, 2, … *N*, resulting in a 3 *N* steps protocol.

The optimisation was performed using a global optimisation scheme, covariance matrix adaptation evolution strategy (CMA-ES, Hansen, 2006) implemented via a Python package PINTS ( Clerx *et al.*, 2019b). All optimisation of the designs were repeated 10 times from different randomly varied initial starting points, and the best designs are presented here. Although we do not expect our design would reach the same global optimum as optimising all > 20 steps at once ( Mirams *et al.*, 2024), our results still show promising protocol designs. We also tried to perform fitting 6-steps-at-once in Mirams *et al.* (2024) and showed that both resulted in similar performance. Finally, the presented results are the optimised results rounded to the nearest one decimal place in millisecond and millivolt for practical implementation ( Mirams *et al.*, 2024).

### Sensitivity-based designs

***Maximising approximated local sensitivity***

For an ion channel current model *I* with *N* parameters *p*_1_, *p*_2_, ... *p _N_*, we define an objective function for each 3-step unit *i* that maximises the absolute value of the sensitivity $\left|\frac{\partial I}{\partial p_{i}}p_{i}\right|$ of the model output *I* with respect to the parameter *p _i_* while minimising all the absolute value of sensitivity of the rest of the parameters. This objective function can be mathematically expressed as


$$S_{i}({\left\{V_{i,j},\Delta t_{i,j}\right\}}_{j=1}^{3})=\frac{\int_{\Delta t_{i,3}}\left|\frac{\partial I}{\partial p_{i}}p_{i}\right|dt}{\Sigma_{k}\int_{\Delta t_{i,3}}\left|\frac{\partial I}{\partial p_{k}}p_{k}\right|dt}.$$

**Figure 5.**
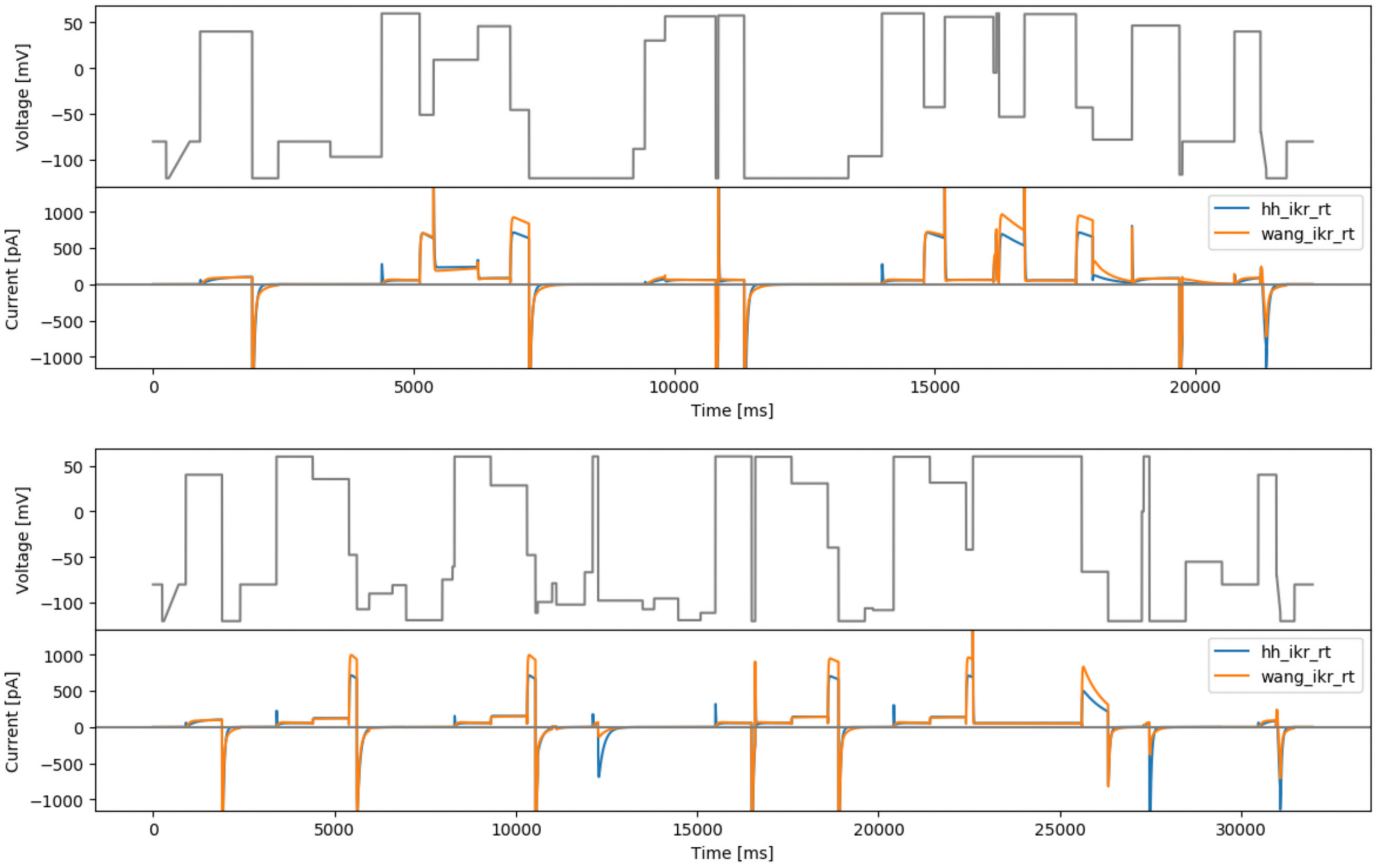
The 3-step local sensitivity designs. Top: protocol based on the Hodgkin-Huxley model (hh3step). Bottom: based on the Wang model (wang3step). With simulated currents from both models shown below the protocols.

The sensitivity was calculated using a first-order central difference scheme with δ *p _i_* being 0.1 % × *p _i_*. Note that the integration is only over the last step of the 3 steps, the idea is to allow the first two steps to vary as much as it would need to be to maximise the approximated local sensitivity across the third step (it is fine if there is low sensitivity because of e.g. full inactivation in the first two steps). This has been repeated for both models and the results are shown in Figure 5.

***Maximising Sobol sensitivity***

Instead of the local sensitivity, we can also replace it with the first-order Sobol global sensitivity indices, given by


$$S_{i}\left({\left\{V_{i,j},\Delta \;t_{i,j}\right\}}_{j=1}^{3}\right)=\frac{1}{\text{Var}(I)}{\text{Var}}_{p_{i}}\left({\text{E}}_{p_{!i}}(I|p_{i})\right).$$

Here the *p _!i_* notation denotes the set of all parameters except *p _i_*. This has been repeated for the Beattie & Wang models. The parameter range ( Table 1) was taken from previous real data fits to staircaseramp, sis, hh3step and wang3step, using the approach from Lei, Clerx, Gavaghan, *et al.* (2019b) without accounting for experimental error ( Lei *et al.*, 2020a; Lei *et al.*, 2020b).

**Figure 6.**
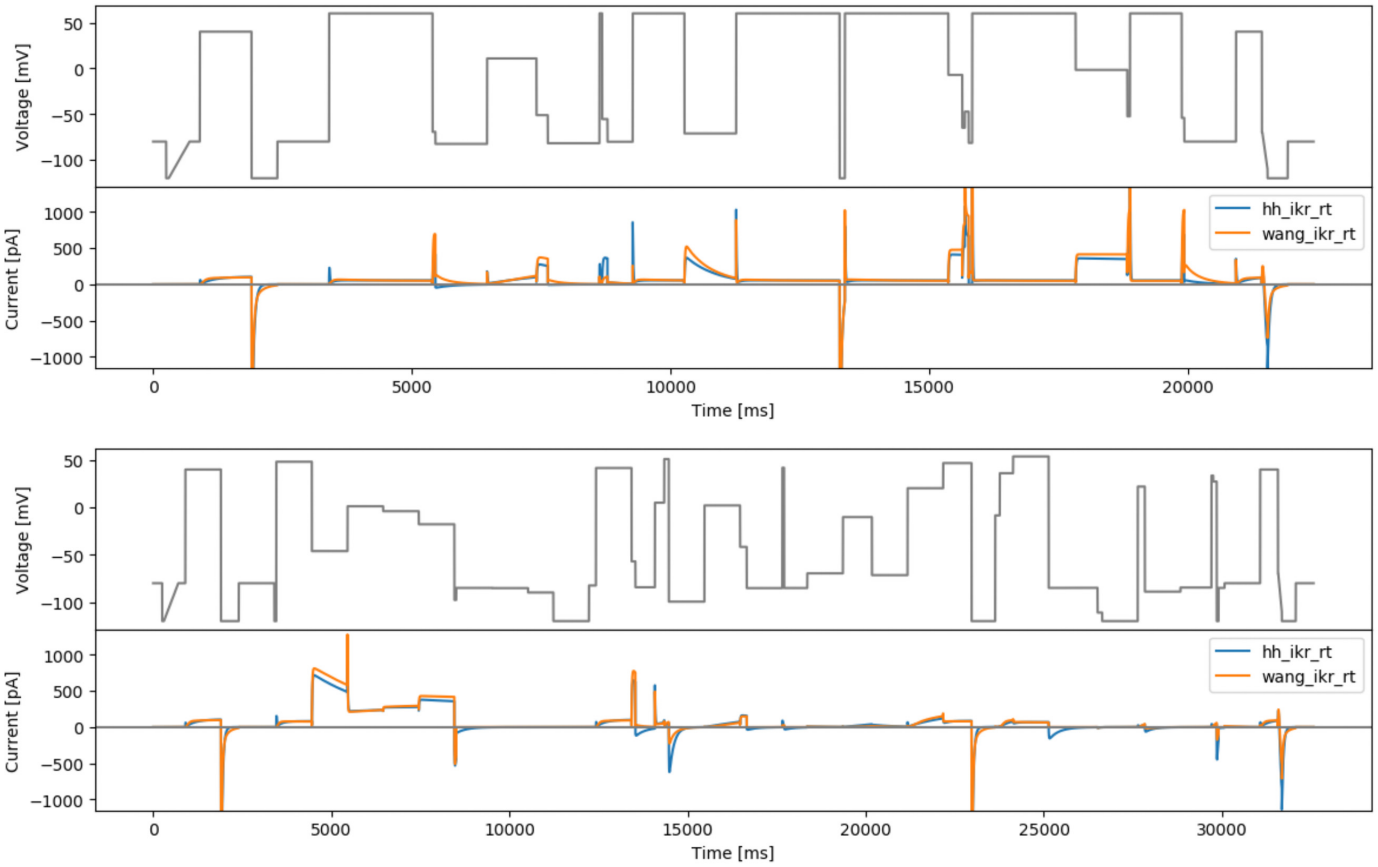
The 3-step Sobol sensitivity protocols. Top: based on the Hodgkin-Huxley model (hhsobol3step). Bottom: based on the Wang model (wangsobol3step). With simulated currents from both models shown below the protocols.

To calculate Sobol sensitivities we used a modified version of the SA-lib library ( Herman & Usher, 2017), to enable easier calculation of sensitivities over time series, which is included in our repository (see Data Availability). The results are shown in Figure 6.

### Gibbs designs

We use the 3-step approach discussed above, but the difference here is that instead of defining each step by two parameters (voltage *V* and duration *Δt*), for each 3-step section we optimise only one of these parameters (either *V* or *Δt*) while randomly picking the other from a uniform distribution. This halves the number of parameters that are inferred to just 3 per 3-step section. However, since we have only the same objective function, all units would return the same optimum (or a few if multi-modal but very limited) which is not desired. Therefore we introduce some stochasticity to the protocol by randomly choosing one of the step parameters and optimising only the other one.

***Maximising model output differences: a brute-force sampling approach***

The approach taken in this design is similar to a global sensitivity analysis. For a given model *I*, we start with randomly picking *M* (ideally ∼ 1000s but practically ∼ 100s of) parameters from model parameter prior, then the objective function to be maximised is the sum of the root mean square deviation (RMSD) values between the model outputs from all combinations of the sampled parameter pairs. The model parameter prior could be an a-priori distribution of the parameters (for example those used in Beattie *et al.*, 2018; Lei *et al.*, 2019b), or based on previous fitting results (see below). The objective function to be maximised for a 3-step unit *i* can be expressed as


$$S({\text{θ}}_{i})=\frac{2}{M^{2}}\sum_{j=1}^{M}\sum_{k>j}^{M}\text{RMSD}\;(I_{j},I_{k}),\;(2)$$

where RMSD( *x*, *y*) denotes the RMSD between *x* and *y*, and *I _j_*, *I _k_* are the model output for the *M* parameter samples. We choose **θ** *_i_* = ${\left\{V_{j}\right\}}_{j=1}^{3}$ with *Δt _j_* ∼ Uniform(50,1000)  ms for odd *i*, and **θ** *_i_* = ${\left\{\Delta \;t_{j}\right\}}_{j=1}^{3}$ with *V _j_* ∼ Uniform(–120,60)  mV for even *i*.

**Figure 7.**
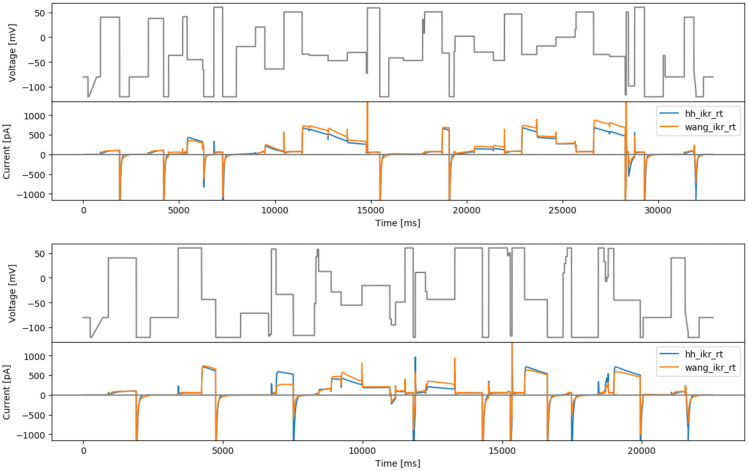
The brute-force sampling protocols. Top: based on the Hodgkin-Huxley model (hhbrute3gstep). Bottom: based on the Wang model (wangbrute3gstep). Simulated currents from both models are shown beneath each protocol.

This has been repeated for the Beattie and Wang models, with the parameter range (prior distribution) was taken from the extremes of the range defined by previous real data fits to staircsaeramp, sis, hh3step and wang3step, as provided in Table 1. The results are shown in Figure 7.

***Maximising differences between two models***

**Figure 8.**
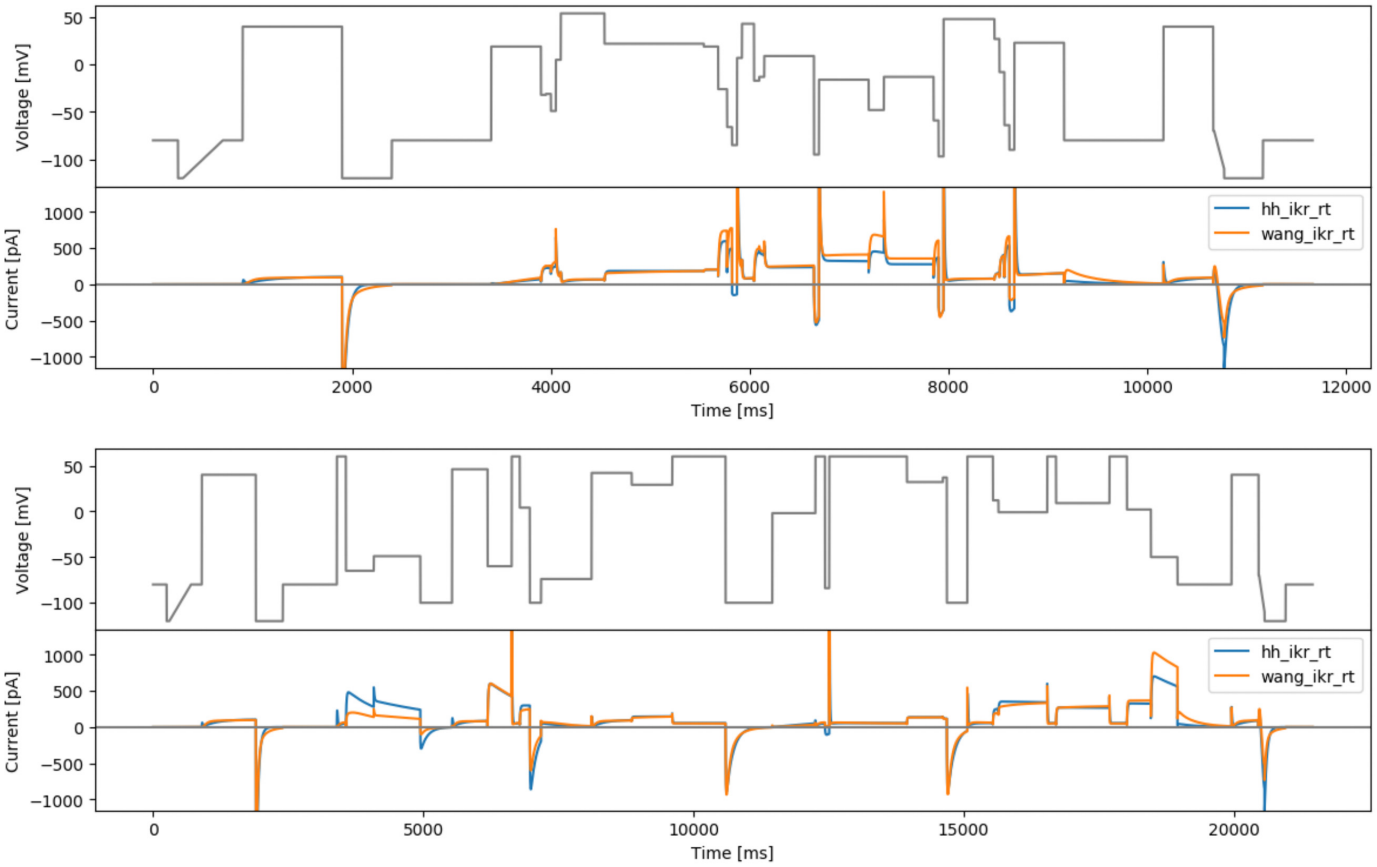
Protocols that maximise the difference between currents from the Beattie and Wang models. Top: based on randomised voltage and optimising time steps (rvotmaxdiff). Bottom: based on randomised time steps and optimised voltages (rtovmaxdiff). Simulated currents from both models are shown beneath each protocol.

Unlike the previously defined approaches, where only one model was involved, this proposed approach aims to distinguish between two candidate models. The objective function is defined as the RMSD value between two model currents, with a given set of model parameters ( Table 1), so it is still a ‘local’ design with respect to model parameters. One protocol randomly picks time parameters for each 3-step unit, and optimises voltages ${\left\{V_{j}\right\}}_{j=1}^{3}$ with *Δt _j_* ∼ Uniform(50,500)  ms and is termed ‘rtovmaxdiff’); and the other method randomly picks voltages and optimises the step durations ${\left\{\Delta \;t_{j}\right\}}_{j=1}^{3}$ with *V _j_* ∼ Uniform(–120,60)  mV, and is known as ‘rvotmaxdiff’. Applying this approach to the Beattie & Wang models results in Figure 8.

### Phase-voltage space filling designs

**Figure 9.**
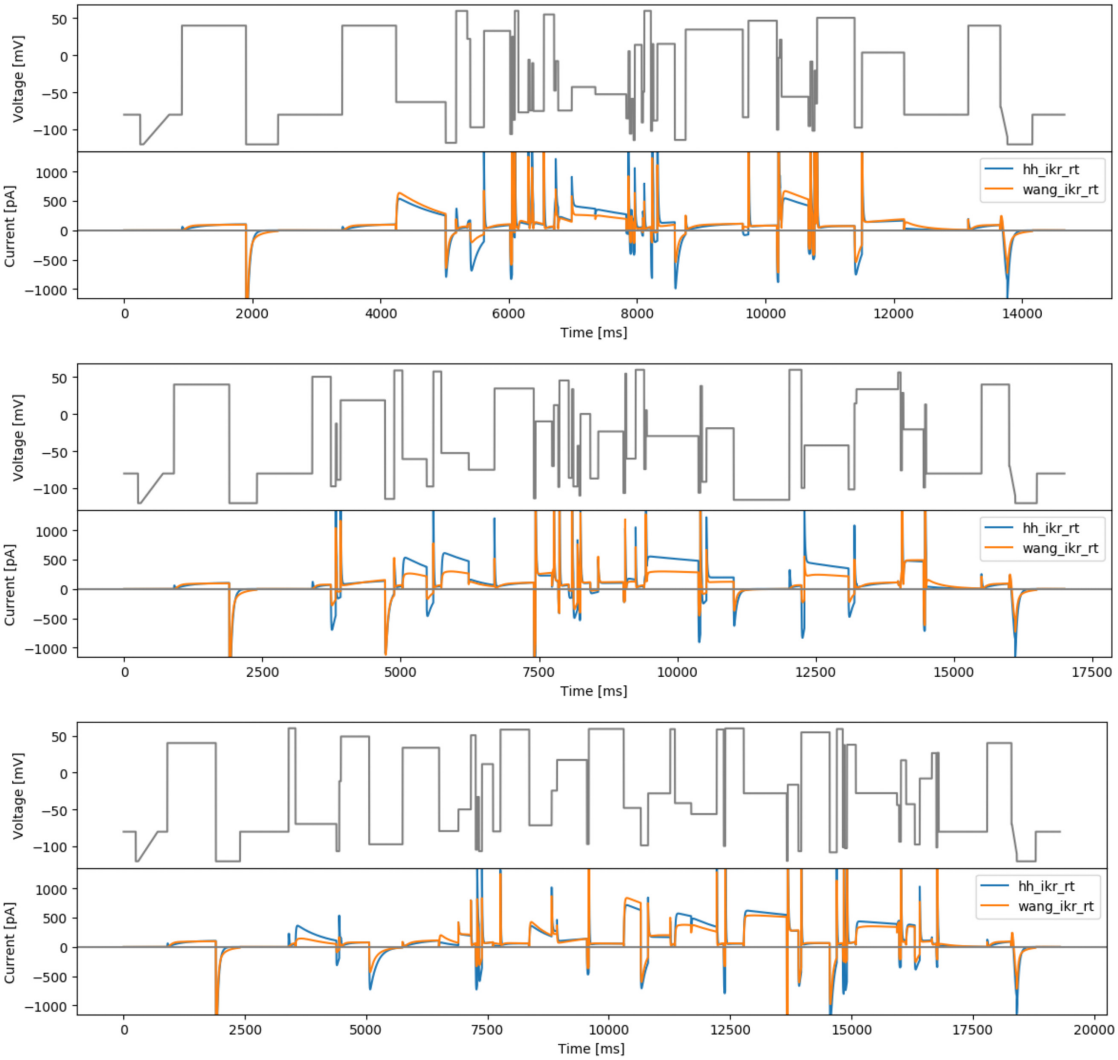
Phase-voltage space filling designs. Top: first phase-voltage space protocol (spacefill26). Middle: second phase-voltage space protocol (spacefill10). Bottom: third phase-voltage space protocol (spacefill19), with simulated currents from both models.

For details of this approach, see Mirams *et al.* (2024). Briefly, an objective function tries to maximise the amount of new boxes that are visited by a model’s trajectory for each new iterative ‘3 step’ set of pulses (as described above) repeating sequentially until we have 17 sets of 3 steps. This approach has a stochastic optimisation step, and produces some protocols that appear to be challenging and information rich, where we appear to have a reasonable amount of current and interesting dynamics. After 30 optimisation runs with different random seeds and initial guesses, we selected the following 3 best protocols based on slightly different criteria:

- Figure 9, top — Number 26: the best space-filling objective function score ( Mirams *et al.*, 2024).
- Figure 9, middle — Number 10: the largest RMSD value between the two models’ simulated currents.
- Figure 9, bottom — Number 19: the best brute-force sampling score ( Eq. (2)) for the Beattie *et al.* (2018) model.

All three protocols visit between 126–132 (58–61%) of the available 216 ‘boxes’ in phase-voltage space. Note that this is a lower percentage than the protocols in Mirams *et al.* (2024) primarily due to 1 ms time samples being used in the 2019 optimisations presented here (see Discussion of Mirams *et al.* (2024)) along with extra initial guesses now being used in the Mirams *et al.* (2024) optimisation procedure to gain slightly higher coverage of the space.

## Automated square waves

Following the same argument as in ‘Maximising differences between two models’ above, this design maximises the differences between two candidate models to aid model selection. Here we use *N* = 3 (as per Beattie *et al.*, 2018) which gives 9 parameters in total (see Equation (1)), with a fixed offset voltage of −30 mV. The square wave parameters are optimised based on an objective function that maximises the RMSD value between two model outputs. As above, the two models have a set of predefined model parameters, so it is still a ‘local’ model parameter method.

**Figure 10.**
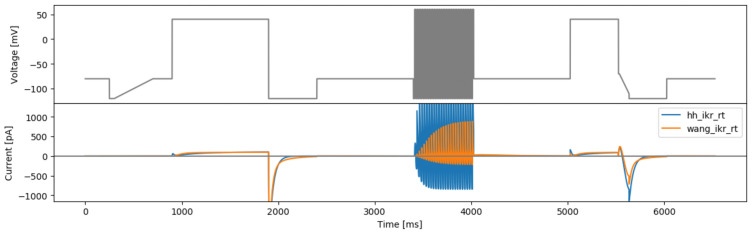
The square wave protocol for maximising two models’ difference (maxdiff) and simulated currents from both models.

This approach was applied to the Beattie and Wang models using their original literature parameters. The resulting protocol ( Figure 10) exhibits extremely high frequency and high amplitude (hitting the boundaries of the protocol parameters) behaviour. We believe these rapid changes of voltage tends to maximise the two model outputs, which is similar to the ‘original sine wave #2’ in Beattie (2015), and is likely to be impractical or uninformative for real experiments.

## Discussion

Developing ion channel models remains a challenging task predominantly due to all the various sources of uncertainty and variability ( Mirams *et al.*, 2016) — in terms of modelling approximations ( Lei *et al.*, 2020c; Lei & Mirams, 2021) as well as experimental noise and artefacts ( Lei *et al.*, 2020a). It is made more difficult due to the sparsity of available data for independent training and validation, with it still being common to calibrate models to all available data ( Whittaker *et al.*, 2020). The protocols presented here encompass many design criteria, including parameterisation, model selection and rigorous testing of the underlying assumptions in hERG models ( Fink & Noble, 2009; Lei *et al.*, 2019b; Mirams *et al.*, 2024). As such, we expect that this collection of voltage clamp protocols will be extremely useful for development of mathematical models for the physiological gating of the hERG potassium channel, and in particular by providing ample validation data for assessing their prediction errors due to model discrepancy ( Shuttleworth *et al.*, 2024).

Experimental high-throughput data on CHO hERG1a cells at room temperature associated with these protocols is available ( Shuttleworth *et al*., 2025). That study also shows the results of fitting the Beattie and Wang models and examines how mathematical hERG model parameter estimates have both a protocol-dependent effect, perhaps associated with model discrepancy as predicted in Shuttleworth *et al*. (2024); and a well-dependent effect, perhaps caused by well-dependent experimental artefacts such as series resistance ( Lei *et al*., 2020a). Further research is ongoing in the best way to account for these effects and adapt protocol design strategies for model selection, particularly for selection of drug binding models ( Patten-Elliott *et al*., 2025).

The same design criteria we have outlined here could easily be applied to other ion channels to create similar suites of protocols, using the provided open source codes.

## Ethics and consent

Ethical approval and consent were not required.

## Data Availability

A spreadsheet of the protocols is available from: https://doi.org/10.6084/m9.figshare.29401097.v1 Licence: CC-BY

## Software availability statement

•    Source code available from: https://github.com/CardiacModelling/protocol-design-hERG

•    Archived software available from: https://doi.org/10.5281/zenodo.13343099 ( Lei *et al.*, 2024).

•    License: BSD 3 clause license
