# Supplementary material for: A range of voltage-clamp protocol designs for rapid capture of hERG kinetics
Source: Wellcome Open Res. 2025 Jul 9;9:673. Originally published 2024 Nov 12. [Version 2] doi: 10.12688/wellcomeopenres.23319.2 (PMC12334913; doi:10.12688/wellcomeopenres.23319.2)
Supplement: Peer review history

# Reviewer report: Reviewer response for version 2

**DOI:** 10.21956/wellcomeopenres.26771.r127448

**Published:** 2025-08-08

**Authors:** David.k Jones

**Affiliations:** 1 University of Michigan, Ann Arbor, USA

The researchers provide a detailed rationale and outline for their protocol. The manuscript is well-written and well-cited, making the details throughout easy to understand and open for further understanding through additional reading of many of the cited papers, both classical and more recent. The authors should be congratulated on a high quality body of work.

Is the rationale for developing the new method (or application) clearly explained?

Yes

Is the description of the method technically sound?

Yes

Are the conclusions about the method and its performance adequately supported by the findings presented in the article?

Yes

If any results are presented, are all the source data underlying the results available to ensure full reproducibility?

Yes

Are sufficient details provided to allow replication of the method development and its use by others?

Yes

Reviewer Expertise:

Ion channel biophysics, cellular and organismal electrophysiology

I confirm that I have read this submission and believe that I have an appropriate level of expertise to confirm that it is of an acceptable scientific standard.

# Reviewer report: Reviewer response for version 2

**DOI:** 10.21956/wellcomeopenres.26771.r127165

**Published:** 2025-08-08

**Authors:** Lucia Romero Perez

**Affiliations:** 1 Universitat Politècnica de València, Valencia, Spain

No further comments to make. The manuscript can go ahead and be indexed.

Is the rationale for developing the new method (or application) clearly explained?

Yes

Is the description of the method technically sound?

Yes

Are the conclusions about the method and its performance adequately supported by the findings presented in the article?

Yes

If any results are presented, are all the source data underlying the results available to ensure full reproducibility?

Yes

Are sufficient details provided to allow replication of the method development and its use by others?

Yes

Reviewer Expertise:

Cardiac modelling

I confirm that I have read this submission and believe that I have an appropriate level of expertise to confirm that it is of an acceptable scientific standard.

# Reviewer report: Reviewer response for version 1

**DOI:** 10.21956/wellcomeopenres.25702.r116930

**Published:** 2025-02-10

**Authors:** Lucia Romero Perez

**Affiliations:** 1 Universitat Politècnica de València, Valencia, Spain

This paper designs short voltage-clamp protocols to get data for hERG ion channel gating with the aim of producing better models of hERG. This is an important topic for cardiac modelling and simulation, and the methodology is sound.

 The results are interesting, although they would be more exciting if there would be experimental data to compare the results provided by the simulations. This could be mentioned in the paper.

 As it is stated “this collection of voltage clamp protocols will be extremely useful for development of mathematical models for the physiological gating of the hERG potassium channels…” However, I guess these protocols produce a lot of data.  From all the simulated protocols in the paper, which ones would you choose if your could only choose two?

 Based on the results produced by the simulations of the paper, what of the two model structures seems to produce more realistic currents?

 I don´t understand the brute-force sampling approach, especially the sentence “For a given model I, we start with randomly picking M (ideally ∼ 1000s but practically ∼ 100s of) parameters from model parameter prior, then the objective function to be optimised is the sum of the root mean square deviation (RMSD) values between the model outputs from all combinations of the sampled parameter pairs.” Does that mean that you try to get a protocol that maximizes de differences in the currents predicted by M different models obtained by changing the values of the parameters buy maintaining the equations? In this case the objective function is maximized, isn`t it?

 If I have understood well, the protocols obtained maximising approximated local sensitivity and Sobol sensitivity produce protocols that contain parts that art especially sensitive to one specific parameter. Could you please indicate what parts are especially sensitive to each parameter in the respective figures?

 It would be better to put the tables with the details of the protocols in an appendix, as they are also illustrated in the figures of the paper.

Is the rationale for developing the new method (or application) clearly explained?

Yes

Is the description of the method technically sound?

Yes

Are the conclusions about the method and its performance adequately supported by the findings presented in the article?

Yes

If any results are presented, are all the source data underlying the results available to ensure full reproducibility?

Yes

Are sufficient details provided to allow replication of the method development and its use by others?

Yes

Reviewer Expertise:

Cardiac modelling

I confirm that I have read this submission and believe that I have an appropriate level of expertise to confirm that it is of an acceptable scientific standard, however I have significant reservations, as outlined above.

# Reviewer report: Reviewer response for version 1

**DOI:** 10.21956/wellcomeopenres.25702.r111463

**Published:** 2024-12-26

**Authors:** Arpad Mike

**Affiliations:** 1 HUN-REN Institute of Experimental Medicine, Budapest, Hungary

Understanding the kinetics of ion channel gating, how it is affected by temperature, and modified by various drugs, is a central question in the pharmacology of ion channels, whether the channel is the primary target, or it is involved in adverse effects. A thorough understanding often requires the construction of a mathematical model of the ion channel. What exact measurements are necessary to create a reliable model is not a trivial problem, and the researchers who created this manuscript are among the pioneers in addressing it. This manuscript investigates how different voltage protocols perform in helping researchers design, test, and compare models of the hERG channel. The methods they propose for designing voltage protocols can be applied for studying other voltage-gated ion channels as well.  

 The methods are solid, and the message is interesting, although the way it is presented could be clearer.

 I have one minor and one major concern with the manuscript.

 The minor issue is the position of Tables 3 to 6.

 The text discusses altogether 18 voltage protocols. The figures follow the order of the text (with the exception of “rtovmaxdiff” and “rvotmaxdiff” protocols, but they are both in Fig. 8, so there is no confusion). The tables, however, follow a rather arbitrary order:

 1-2-3-4-5__13-18-6__7-8-9-10-15__16-17-11-12-14

 What was the reason for this? The text is disrupted by several pages of large tables(they take up one-third of the manuscript), but they are not even in the right order. I suggest placing the tables describing all protocols (in the correct order) at the end of the text. I would even consider putting them in a single supplemental file.

 My major concern regards the fundamental “So what?” question. Who will be the readers, and why should they be interested?

 (By the way, the changes I suggest below are not intended as requirements for acceptance, but rather recommendations for improvement, which the authors may accept if they agree.)

 I believe the primary audience of this particular paper would be pharmacologists, and the motivation for reading this manuscript would be, that one tries to reach a more profound understanding of the effect of a certain compound on a certain ion channel, or on a set of different ion channels.

 Because the prospective readers are the type that seeks to understand things, I suggest that authors should discuss these results from a less technical and more practical point of view. It is nice that  automatically designed protocols can differentiate between models, but which of the two models reproduced actual channel kinetics better? Does the topology of models tell something about which actual conformational transitions occur during the operation of the channel? Might it be necessary to design a model with a completely new topology? What would be the simplest (not the best) protocol that can differentiate between model topologies?

 From a pharmacologist’s point of view, finding the model that best reproduces ion channel gating is only the first step, and it cannot be separated from the next one: finding a model that best reproduces drug effects on that specific ion channel. I understand that addressing this second question would exceed the limits of this manuscript, and I am aware that the authors have already dealt with the problem of modeling drug effects ( *e.g.* in their BJP 2023 paper), but not from this aspect: What are the best protocols that can help determine drug mechanisms of action?

 If they intend to pursue this goal, I am not sure that the “find the best protocol that can fit into 51 segments” approach is the best one. I would also suggest the “find the simplest protocol that still can differentiate between models” approach. The brute force, local or global sensitivity, space-filling, etc. designs are impressive, but they may not be as widely implemented by pharmacologist. In contrast, protocols like the “staircase”, or even something like the “sis” do make sense. Pharmacologists like to design their own protocols, and like to be able to justify why it looks the way it does.

 In summary, the manuscript is a valuable collection of strategies for designing efficient voltage protocols for the study of hERG channels. I would personally prefer a more analytical, explanatory discussion of specific elements of the protocols, rather than focusing primarily on the results of automated processes.

Is the rationale for developing the new method (or application) clearly explained?

Yes

Is the description of the method technically sound?

Yes

Are the conclusions about the method and its performance adequately supported by the findings presented in the article?

Yes

If any results are presented, are all the source data underlying the results available to ensure full reproducibility?

Yes

Are sufficient details provided to allow replication of the method development and its use by others?

Yes

Reviewer Expertise:

pharmacology of ion channels

I confirm that I have read this submission and believe that I have an appropriate level of expertise to confirm that it is of an acceptable scientific standard.
